# The effectiveness and safety of noninvasive brain stimulation technology combined with speech training on aphasia after stroke: A systematic review and meta-analysis

**DOI:** 10.1097/MD.0000000000036880

**Published:** 2024-01-12

**Authors:** Congli Han, Jiqin Tang, Bingshun Tang, Tao Han, Jienuo Pan, Nan Wang

**Affiliations:** a College of Rehabilitation Medicine, Shandong University of Traditional Chinese Medicine, Jinan, Shandong, China; b The First Clinical Medical College of Shandong University of Traditional Chinese Medicine, Jinan, Shandong, China; c College of Traditional Chinese Medicine, Shandong University of Traditional Chinese Medicine, Jinan, Shandong, China.

**Keywords:** meta-analysis, non-invasive brain stimulation, post-stroke aphasia, speech training

## Abstract

**Background:**

Although the effectiveness of noninvasive brain stimulation (NIBS) technology in assisting rehabilitation is widely recognized, its therapeutic efficacy in patients with poststroke aphasia (PSA) requires further validation. Here, we aimed to explore the efficacy and safety of the NIBS technique combined with speech training in PSA by traditional Meta-analysis and to compare the intervention effects of the 2 NIBS techniques by Network meta-analysis.

**Methods:**

Randomized controlled trials of the NIBS technique combined with speech training for treating PSA in 9 databases, including Web of Science, PubMed, and CNKI, and 2 clinical trial registries were searched by computer. Literature screening was performed using EndNote X9 software, and data analysis and presentation of results were performed using RevMan 5.4.1 and Stata 17.0 software.

**Results:**

Screening yielded 17 studies with 1013 patients with PSA. Meta-analysis showed that aphasia quotient scores were higher in the intervention group than in the control group [standardized mean difference (SMD) = 1.06, 95% confidence interval (CI) (0.63, 1.49), *Z* = 4.80, *P* < .00001]; Western aphasia battery scores on all 4 subscales were higher than those of the control group, the spontaneous language score is [SMD = 0.62, 95% CI (0.46, 0.78), *Z* = 7.52, *P* < .00001], the listening comprehension score is [SMD = 0.46, 95% CI (0.30, 0.62), *Z* = 5.62, *P* < .00001], the repetition score is [SMD = 1.14, 95% CI (0.59, 1.70), *Z* = 4.04, *P* < .0001], the naming score is [SMD = 1.06, 95% CI (0.79, 1.32), *Z* = 7.85, *P* < .00001]; The effective rate of the intervention group was higher than that of the control group [odd ratio = 4.19, 95% CI (2.39, 7.37), *Z* = 4.99, *P* < .00001]. The results of the Network meta-analysis showed that the best probability ranking of the 2 NIBS techniques combined with speech training in improving aphasia quotient scores was repetitive transcranial magnetic stimulation group (92.2%) > transcranial direct current stimulation group (55.7%). Regarding safety, it was not found that the NIBS technique combined with speech training to treat PSA increases the risk of adverse reactions.

**Conclusion:**

The NIBS technique combined with speech training can effectively improve the recovery of language function in PSA patients with minimal adverse effects, and the clinic can give priority to r TMS combined with speech training in treating PSA.

## 1. Introduction

Poststroke aphasia (PSA) is an acquired language disorder caused by damage to the dominant hemisphere speech area as a result of stroke.^[1]^ According to statistics, about 32 percent of adult stroke patients suffer from aphasia and are unable to communicate normally, which seriously affects the prognosis and quality of life of patients.^[2]^ Speech training is the foundation of speech rehabilitation in PSA patients. In past clinical practice, speech therapists usually used conventional speech training methods such as Schuell stimulation therapy, music therapy, and constraint-induced language therapy to improve the speech dysfunction of PSA patients. However, there are some limitations in using speech training alone for treatment. As speech training mainly relies on professional speech therapists to conduct 1-on-1 training, the duration of a single training session is relatively short. Patients must continue to undergo boring speech training for a long, which may lead to resistance and negatively affect the therapeutic effect. Since the current treatment effect of speech training alone is not satisfactory enough, it is therefore considered that other treatment methods should be added to the speech training to enhance the efficacy of the treatment.

In recent years, noninvasive brain stimulation (NIBS) technology has been widely used in treating various neurological disorders, including stroke, Parkinson, and vascular dementia, due to its advantages of noninvasiveness, safety, and high efficiency.^[3,4]^ Repetitive transcranial magnetic stimulation (rTMS) and transcranial direct current stimulation (tDCS) are both specific applications of the NIBS technique. Their most important mechanism of action is to regulate the balance between the 2 cerebral hemispheres, increase the excitability of the dominant hemisphere, or inhibit the excitability of the nondominant hemisphere.^[5]^ Studies have shown that the combination of NIBS techniques based on speech training is expected to promote the recovery of speech function in PSA patients.^[6]^ Although the results of several therapeutic studies of the NIBS technique combined with speech training for patients with PSA have generally been promising,^[7,8]^ there have also been studies showing no significant therapeutic effect after intervention with NIBS combined with speech training.^[9]^ It is yet to be possible to determine whether the combination of NIBS techniques and speech training is more effective due to the small-sample sizes of individual studies and differences in measurement methods and study designs.^[10]^ In addition, there are fewer comparative studies of the 2 NIBS techniques, r TMS and t DCS, in combination with speech training for treating PSA, which is not conducive to optimal decision-making by rehabilitation therapists. Therefore, this study used Meta-analysis to explore the effect of the NIBS technique combined with speech training in the treatment of PSA and directly or indirectly compared the intervention effect of the 2 NIBS techniques through Network meta-analysis, aiming to provide an evidence-based basis for clinical practice and to open up clinical treatment ideas.

## 2. Methods

We strictly followed the guidelines of the PRISMA^[11]^ and registered in the PROSPERO platform, the ID Number is CRD42023408998 (https://www.crd.york.ac.uk/PROSPERO/). Ethical approval is not required because the information used in this study is obtained from published randomized controlled trials (RCTs).

### 2.1. Eligibility criteria

We searched PubMed, Embase, Cochrane, Web of Science, OVID, CNKI, Wanfang, CBM, VIP, and Clinical Trials, and China Clinical Trials Registry from the establishment of the database to August 25, 2023. At the same time, we used a combination of Medical Subject Headings and free-text search terms to adjust the retrieval strategy based on the retrieval characteristics of each database. The following search terms were used to search in the English database: “stroke,” “ischemic stroke,” “cerebral infarction,” “aphasia,” “dysphasia,” “poststroke aphasia,” “language disorders,” “noninvasive brain stimulation,” “repetitive transcranial magnetic stimulation,” “transcranial direct current stimulation,” “randomized controlled trial”. Equivalent search terms were used for the Chinese databases. In addition, searches were conducted in the references of the included literature and in published systematic reviews to ensure that all relevant literature was retrieved.

### 2.2. Inclusion and exclusion criteria

#### 2.2.1. Types of studies.

RCTs, only studies in Chinese and English will be included, but not limited to country and publication status.

#### 2.2.2. Types of participants.

Patients who met the 4th National Conference on Cerebrovascular Disease^[12]^ or other relevant diagnostic criteria were diagnosed with PSA through the aphasia screening tool.

#### 2.2.3. Types of intervention and comparators.

The intervention group was given the NIBS combined with speech training, and the control group was given speech training or sham stimulation combined with speech training.

#### 2.2.4. Types of outcome measures.

The primary outcome measure was the aphasia quotient (AQ). The secondary outcome measures were efficiency, adverse reaction, and Western aphasia battery (WAB), including spontaneous speech, listening comprehension, repetition, and naming.

#### 2.2.5. Exclusion criteria.

1. Non-RCTs, such as reviews, conference summaries, cell or animal experiments.
2. The interventions were not consistent with the requirements of this study.
3. The resulting data were incomplete, could not be converted, or there were errors.
4. Repeat publication.
5. There were no relevant outcome indicators.

### 2.3. Literature screening and data extraction

After completing the preliminary literature search, we imported the obtained literature into the EndNote X9 software for management. Literature data for inclusion in the final decision will be extracted using a pre-developed table. Extracting information includes the following: first author, publication time, sample size, age, intervention, stimulation intensity, intervention time, and outcome indicators.

In this process, to ensure the accuracy of the data, it is necessary to cross-check the data after the 2 researchers’ completion. If there are different opinions, they will be discussed, and a consensus will finally be reached.

### 2.4. Methodological quality evaluation of inclusion studies

Two reviewers will independently assess the risk of bias according to The Risk of Bias 2 tool for randomized trials in the Cochrane Handbook, based on the following domains: Randomization process; Deviations from intended interventions; Missing outcome data; Measurement of the outcome; Selection of the reported result. Furthermore, each item is classified into 3 levels: “low risk,” “high risk,” and “some concerns”.^[13]^ Any inconsistencies in the assessment results will be resolved through discussion with a third party.

Two researchers will independently use the Grading of Recommendations Assessment, Development, and Evaluation (GRADE, http://gradepro.org/) system to assess the quality of the direct and indirect evidence for this study. The quality of evidence will be graded into 4 levels: very low, low, moderate, or high.^[14]^

### 2.5. Statistical analysis

In this study, we used RevMan 5.4.1 software (https://tech.cochrane.org/revman) for meta-analysis. Standardized mean difference (SMD) and 95% confidence interval (CI) were selected as effect indicators for continuous variables, and odds ratio and 95% CI were selected as effect indicators for dichotomous variables. The chi-square test was used to perform heterogeneity analysis. If *P* > .1 and *I²* ≤ 50%, it indicates no heterogeneity between the studies, and a fixed effects model was used. Otherwise, it indicates significant heterogeneity between the individual studies, and the analysis was performed using a random effects model. If heterogeneity existed, sensitivity or subgroup analysis was required to find the source of heterogeneity.

Evidence network diagrams were drawn using Stata 17.0 software. When closed loops existed in the evidence network diagrams, the degree of consistency between the results of the direct and indirect comparisons needed to be assessed through the inconsistency test; vice versa, the consistency model was used for statistical analysis. The Network meta-analysis results are presented through a 2-by-2 comparative forest plot, with SMD and 95% CI selected as indicators of the effect of continuous variables. The 2 NIBS techniques were ranked according to the surface under the cumulative ranking (SUCRA). In addition, corrected-comparison funnel plots were used to test for publication bias and small-sample effects when the number of included studies for the outcome indicator was ≥ 10.

## 3. Results

### 3.1. Literature search results

**Figure 1.**
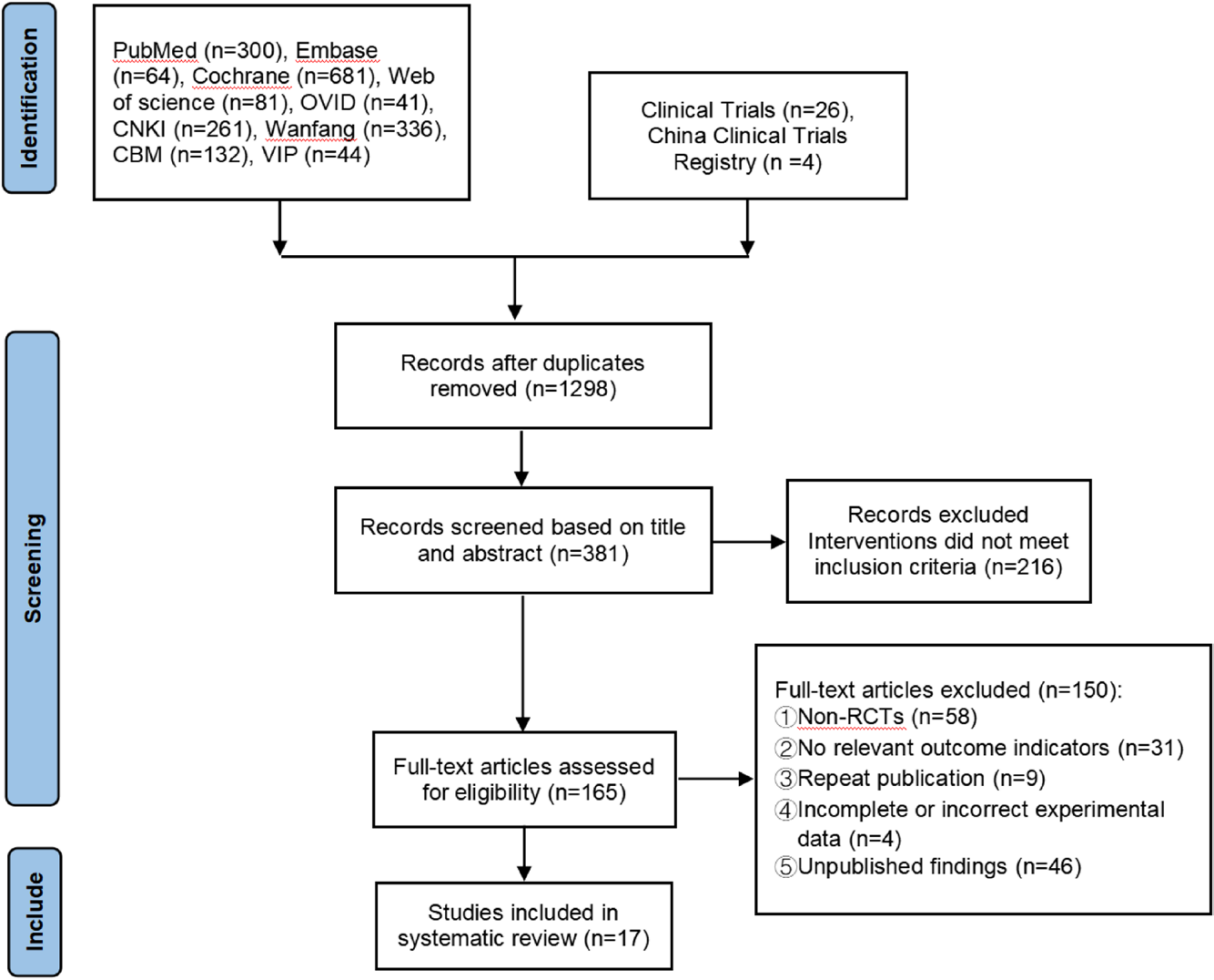
Flow chart of literature screening.

A total of 1970 documents were initially retrieved, and 17 studies were included after 3 screening processes: weight removal, reading the title and abstract, and reading the full text. The literature screening process is shown in Figure 1.

### 3.2. Basic characteristics of the included studies

All 17 studies were 2-arm clinical studies involving 1013 patients with PSA. The year of publication of the included literature was ranged from 2017 to 2022. Among these, 10 studies^[15–24]^ used rTMS + speech training (ST) for treatment, and 7^[25–31]^ used tDCS + ST. The basic information of the literature is shown in Table 1. The NIBS technology treatment protocols for each study are shown in Table 2.

**Table 1:** Basic information on literature.

Study	Sample size (E/C)	Average age (yr)		Type of aphasia	Intervention measure		Outcome indicators
		E	C		E	C	
Li ZH 2018^[21]^	15/15	65.3 ± 5.6	68.3 ± 5.8	Broca aphasia	rTMS + ST	SS + ST	②③
Shen S 2018^[22]^	30/30	57.31 ± 2.51	57.28 ± 2.35	NR	rTMS + ST	ST	①
Fan CP 2017^[17]^	25/25	NR		NR	rTMS + ST	ST	②
Cui C 2019^[18]^	36/36	43.29 ± 1.27	44.23 ± 1.31	Non-fluent aphasia	rTMS + ST	ST	①②③
Gu HP 2019^[19]^	50/50	65.69 ± 7.21	67.30 ± 6.51	NR	rTMS + ST	ST	②
Liu C 2021^[20]^	40/40	54.1 ± 6.2	53.3 ± 5.4	NR	rTMS + ST	ST	②③
Zhou HY 2021^[23]^	53/53	61.25 ± 8.41	59.87 ± 7.64	Broca aphasia	rTMS + ST	ST	①②③
Haghighi, M.2018^[15]^	6/6	NR		Broca aphasia	rTMS + ST	SS + ST	②③
Bai, G. 2022^[16]^	30/30	63.47 ± 7.81	59.91 ± 8.58	Non-fluent aphasia	rTMS + ST	SS + ST	②③
Yoon, T.H. 2015^[24]^	10/10	60.46 ± 9.63	61.13 ± 8.72	Non-fluent aphasia	rTMS + ST	ST	②③
Zhao, Q. 2021^[25]^	8/10	58.00 ± 8.718		non-fluent aphasia	tDCS + ST	SS + ST	②③
Wang L 2018^[27]^	21/21	54 ± 11.524	53.14 ± 10.641	NR	tDCS + ST	ST	②
Tao YY 2019^[28]^	16/15	51.31 ± 14.07	43.53 ± 9.44	Fluency aphasia in 16 cases; non-fluent aphasia in 15cases	tDCS + ST	ST	②
Pan WY 2021^[30]^	48/48	56.8 ± 10.3	56.1 ± 9.5	Broca aphasia	tDCS + ST	ST	①
Zhang Q 2020^[29]^	50/50	65.07 ± 3.42	64.79 ± 2.55	NR	tDCS + ST	ST	①③
Zhang H 2017^[26]^	18/18	59 ± 6	55 ± 8	Non-fluent aphasia	tDCS + ST	SS + ST	②③
Li C 2022^[31]^	50/50	50.56 ± 5.28	50.22 ± 5.12	NR	tDCS + ST	ST	②③

① = efficient, ② = aphasia quotient, ③ = western aphasia battery.

C = control group, E = experimental group, NR = not reported, rTMS+ST = repetitive transcranial magnetic stimulation + speech training, SS + ST = sham stimulation + speech training, ST = speech training, tDCS+ST = transcranial direct current stimulation + speech training.

**Table 2:** NIBS technical treatment program.

Study	NIBS technique	Stimulus site	Stimulus parameter	Intervention time
Li ZH 2018^[21]^	rTMS	Broca mirror area in the back of the right inferior frontal gyrus	1 Hz, 80% MEP, 1200 pulses/d	5 d/w, 3 w
Shen S 2018^[22]^	rTMS	Broca mirror area in the back of the right inferior frontal gyrus	0.5 Hz, 80% MEP, 600 pulses/d	5 d/w, 4 w
Fan CP 2017^[17]^	rTMS	NR	1 Hz, 90% MEP, 1200 pulses/d	5 d/w, 4 w
Cui C 2019^[18]^	rTMS	NR	1 Hz, 90% MEP, 1200 pulses/d	5d/w, 8 w
Gu HP 2019^[19]^	rTMS	Back of the right superior temporal gyrus	1 Hz, 80% MEP, 1200 pulses/d	5 d/w, 4w
Liu C 2021^[20]^	rTMS	Left Broca and Wernicke areas	10 Hz, 90% MEP, 1200 pulses/d	5 d/w, 4 w
Zhou HY 2021^[23]^	rTMS	Broca mirror area in the back of the right inferior frontal gyrus	1 Hz, 90% MEP, 1200 pulses/d	5 d/w, 4 w
Haghighi, M.2018^[15]^	rTMS	Broca mirror area in the back of the right inferior frontal gyrus	1 Hz, 100% MEP, 30 min/d	5 d/w, 2 w
Bai, G. 2022^[16]^	rTMS	Broca mirror area in the back of the right inferior frontal gyrus	1 Hz, 80% MEP, 1,000 pulses/d	5 d/w, 4 w
Yoon, T.H. 2015^[24]^	rTMS	Right subfrontal gyrus	1 Hz, 90% MEP, 1200 pulses/d	5 d/w, 4 w
Zhao, Q. 2021^[25]^	tDCS	The anode is placed in the Broca area of the left inferior frontal gyrus, and the cathode is placed in the right shoulder	2 mA, 20 min/d	5 d/w, 4 w
Wang L 2018^[27]^	tDCS	The anode is placed in the Broca area of the left inferior frontal gyrus, and the cathode is placed in the right shoulder	1.1 mA, 20 min/d	5 d/w, 2 w
Tao YY 2019^[28]^	tDCS	The anode was placed in the L-IFG body surface area of the head, and the cathode was placed on the right shoulder	1.5 mA, 20 min/d	5 d/w, 2 w
Pan WY 2021^[30]^	tDCS	Anodic stimulation: anode on the left Broca area, cathode on the right shoulder Cathodic stimulation: anode on the left shoulder, cathode on the right Broca mirror area	Anodic stimulation: 1.2 mA, 20 min/d Cathodic stimulation: 1.2 mA, 20 min/d	6 d/w, 6 w
Zhang Q 2020^[29]^	tDCS	Anodic stimulation: anode on the left Broca area, cathode on the right shoulder Cathodic stimulation: anode on the left shoulder, cathode on the right Broca mirror area	Anodic stimulation: 1.2 mA, 20 min/d Cathodic stimulation: 1.2 mA, 20 min/d	6 d/w, 6w
Zhang H 2017^[26]^	tDCS	Anodic stimulation: anode on the left Broca area, cathode on the right shoulder Cathodic stimulation: anode on the left shoulder, cathode on the right Broca mirror area	Anodic stimulation: 1.2 mA, 20 min/d Cathodic stimulation: 1.2 mA, 20 min/d	6 d/w, 5 w
Li C 2022^[31]^	tDCS	Anodic stimulation: anode on the left Broca area, cathode on the right shoulder Cathodic stimulation: anode on the left shoulder, cathode on the right Broca mirror area	Anodic stimulation: 1.2 mA, 20 min/d Cathodic stimulation: 1.2 mA, 20 min/d	6 d/w, 5 w

MEP = motor-evoked potential, NIBS = noninvasive brain stimulation, NR = not reported, rTMS = repetitive transcranial magnetic stimulation, tDCS = transcranial direct current stimulation.

### 3.3. Literature quality assessment

**Figure 2.**
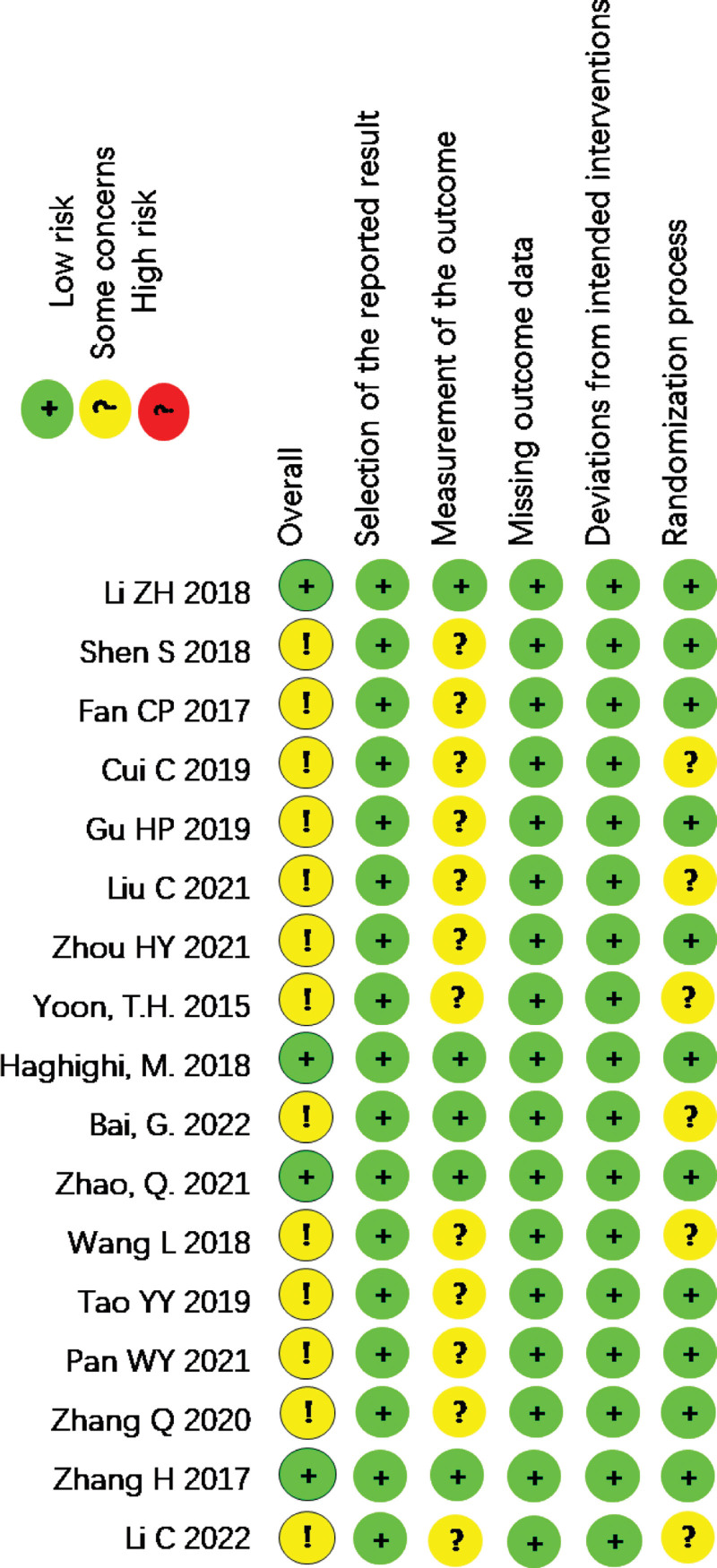
Summary of risk of bias for included studies.

All 17 studies were RCTs, of which 4^[15,21,26,29]^ had a low overall risk of bias, and the remaining had a medium overall risk. Regarding randomization, 11 studies^[17,19–23,26,29–31]^ used the random number table method, 2 studies^[15,25]^ used the opaque letterbox/envelope method, and the remaining 4 studies^[16,19,23,27]^ did not report a specific randomization process. Only 3 studies^[15,21,25]^ reported implementing allocation concealment and blinding. Data from all studies were complete, and no selective reporting of results and other risks of bias were identified. The results of the risk of bias evaluation are shown in Figure 2.

### 3.4. Meta-analysis results

#### 3.4.1. AQ scores.

**Figure 3.**
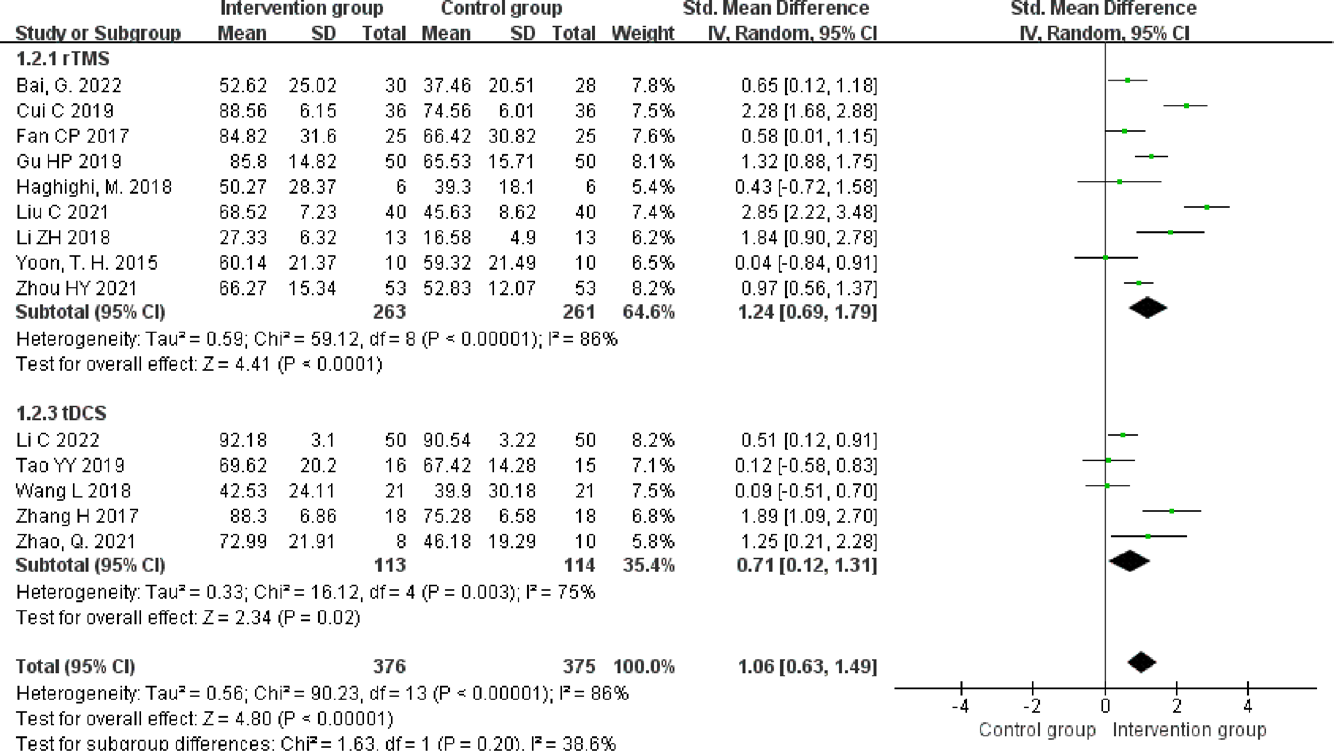
Forest plot of meta-analysis of AQ scores. AQ = aphasia quotient.

A total of 14 studies^[15–21,23–28,31]^ reported AQ scores, and the heterogeneity result was *P* < .00001, *I^2^* = 86%, so it was analyzed using the random effects model. The results showed that AQ scores were higher in the NIBS + ST group than in the control group, and the difference was statistically significant (SMD = 1.06, 95% CI [0.63, 1.49], *Z* = 4.80, *P* < .00001). Subgroups were analyzed according to intervention. The results showed that AQ scores were higher in the rTMS + ST group than in the control group, and the difference was statistically significant (SMD = 1.24, 95% CI [0.69, 1.79], *Z* = 4.41, *P* < .0001). AQ scores were higher in the tDCS + ST group than in the control group, and the difference was statistically significant (SMD = 0.71, 95% CI [0.12, 1.31], *Z* = 2.34, *P* = .02), see Figure 3.

#### 3.4.2. WAB-Spontaneous speech.

**Figure 4.**
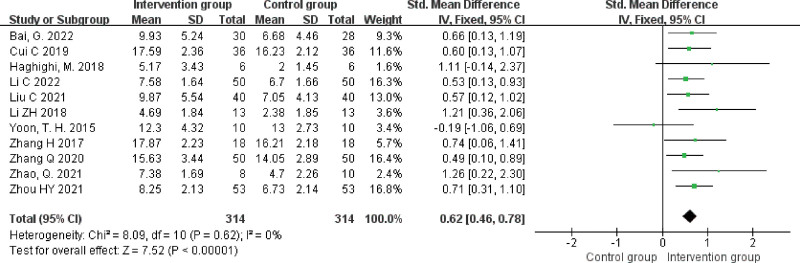
Forest plot of meta-analysis of spontaneous speech scores.

A total of 11 studies^[15,16,18,20,21,23–26,29,31]^ reported spontaneous speech scores with a heterogeneity result of *P* = .62, *I^2^* = 0%, analyzed using a fixed effects model. The results showed that spontaneous speech scores were higher in the NIBS + ST group than in the control group, and the difference was statistically significant (SMD = 0.62, 95% CI [0.46, 0.78], *Z* = 7.52, *P* < .00001), see Figure 4.

#### 3.4.3. WAB-Listening comprehension scores.

**Figure 5.**
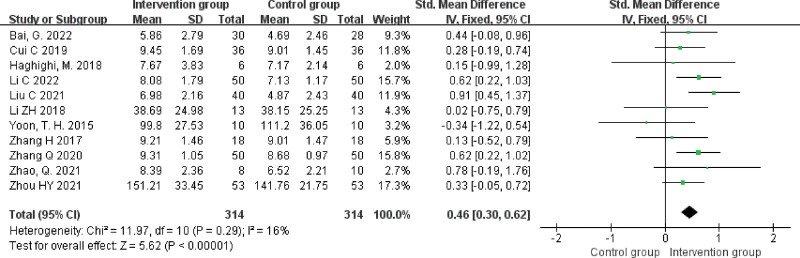
Forest plot of meta-analysis of listening comprehension scores.

A total of 11 studies^[15,16,18,20,21,23–26,29,31]^ reported listening comprehension scores with a heterogeneity result of *P *= .29, *I^2^* = 16%, and a fixed effects model was chosen for analysis. The results showed that PSA patients in the NIBS + ST group had higher listening comprehension scores than those in the control group, and the difference was statistically significant (SMD = 0.46, 95% CI [0.30, 0.62], *Z* = 5.62, *P* < .00001), see Figure 5.

#### 3.4.4. WAB-repetition scores.

**Figure 6.**
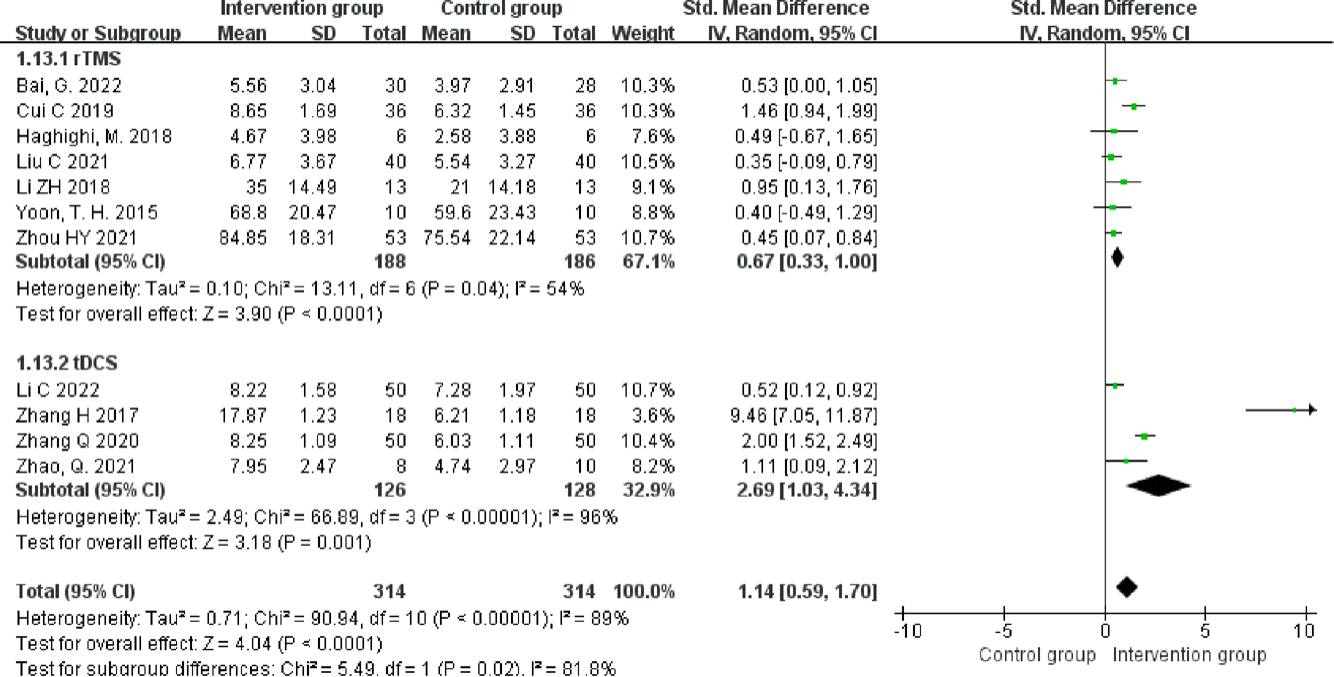
Forest plot of meta-analysis of repetition scores.

A total of 11 studies^[15,16,18,20,21,23–26,29,31]^ reported retelling scores with a heterogeneity result of *P* < .00001, *I^2^* = 89%, and were selected for random effects model analysis. The results showed that PSA patients in the NIBS + ST group had higher restatement scores than those in the control group, and the difference was statistically significant (SMD = 1.14, 95% CI [0.59, 1.70], Z = 4.04, *P* < .0001). Subgroup analyses were performed according to the intervention. The results showed that the rTMS + ST group had higher repetition scores than the control group, with a statistically significant difference (SMD = 0.67, 95% CI [0.33, 1.00], Z = 3.90, *P* < .0001); the tDCS + ST group had higher repetition scores than the control group, with a statistically significant difference (SMD = 2.69, 95% CI [1.03, 4.34], *Z* = 3.18, *P* = .001), see Figure 6.

#### 3.4.5. WAB-naming scores.

**Figure 7.**
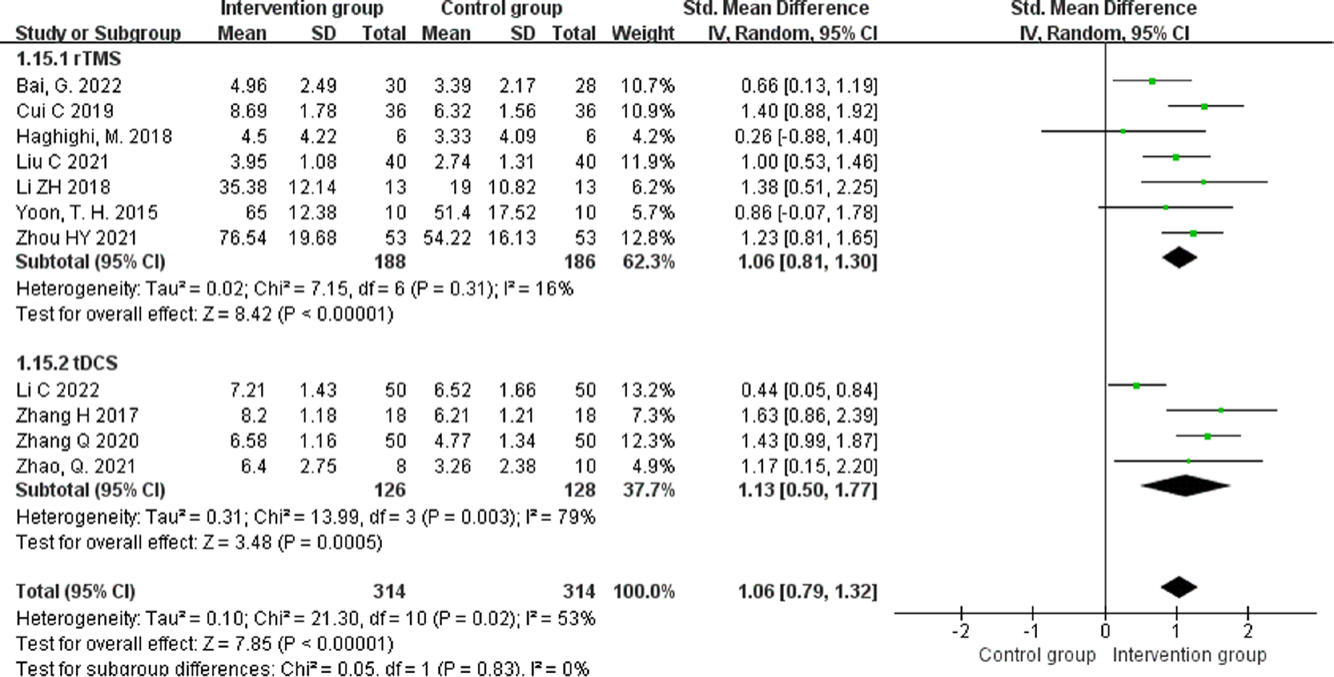
Forest plot of meta-analysis of naming scores.

A total of 11 studies^[15,16,18,20,21,23–26,29,31]^ reported naming scores with a heterogeneity result of *P* = .02, *I^2^* = 53%, analyzed using a random effects model. The results showed that the NIBS + ST group had higher naming scores than the control group, and the difference was statistically significant (SMD = 1.06, 95% CI [0.79, 1.32], *Z* = 7.85, *P* < .00001). Subgroup analyses were performed according to the intervention. The results showed that naming scores were higher in the rTMS + ST group than in the control group, with a statistically significant difference (SMD = 1.06, 95% CI [0.81, 1.30], *Z* = 8.42, *P* < .0001), and naming scores were higher in the tDCS + ST group than in the control group, with a statistically significant difference (SMD = 1.13, 95% CI [0.50, 1.77], *Z* = 3.48, *P* = .0005), see Figure 7.

#### 3.4.6. Clinical efficiency.

**Figure 8.**
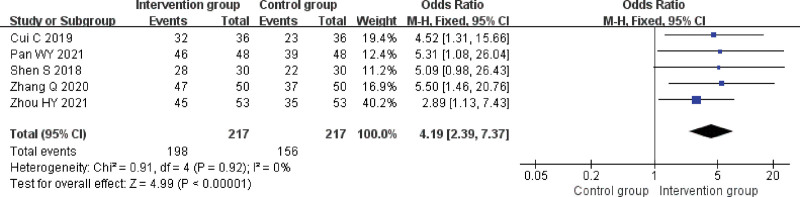
Forest plot for efficient meta-analysis.

A total of 5 studies^[18,22,23,29,30]^ reported the efficacy rate with heterogeneity results of *P* = .92, *I^2^* = 0% analyzed using a fixed effects model. The results showed that the clinical effectiveness rate was higher in the NIBS + ST group than in the control group, and the difference was statistically significant (odd ratio = 4.19, 95% CI [2.39, 7.37], *Z* = 4.99, *P* < .00001), see Figure 8.

### 3.5. Network meta-analysis results

**Figure 9.**
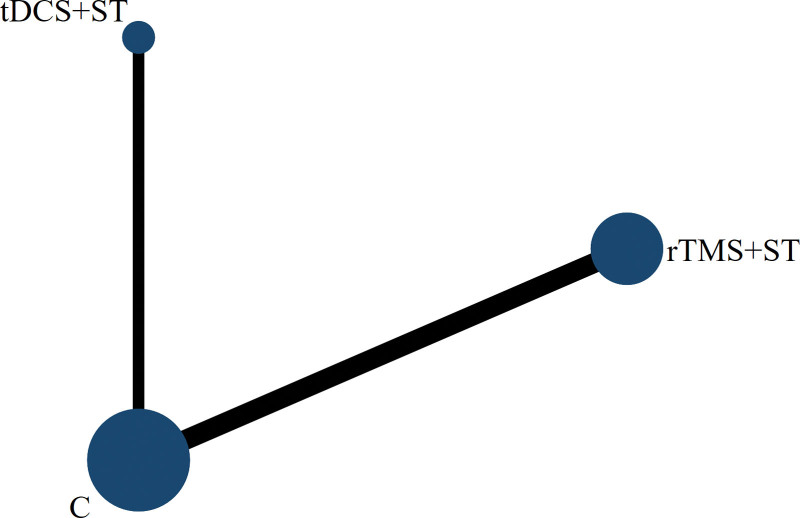
Network evidence map (C = control group).

**Figure 10.**
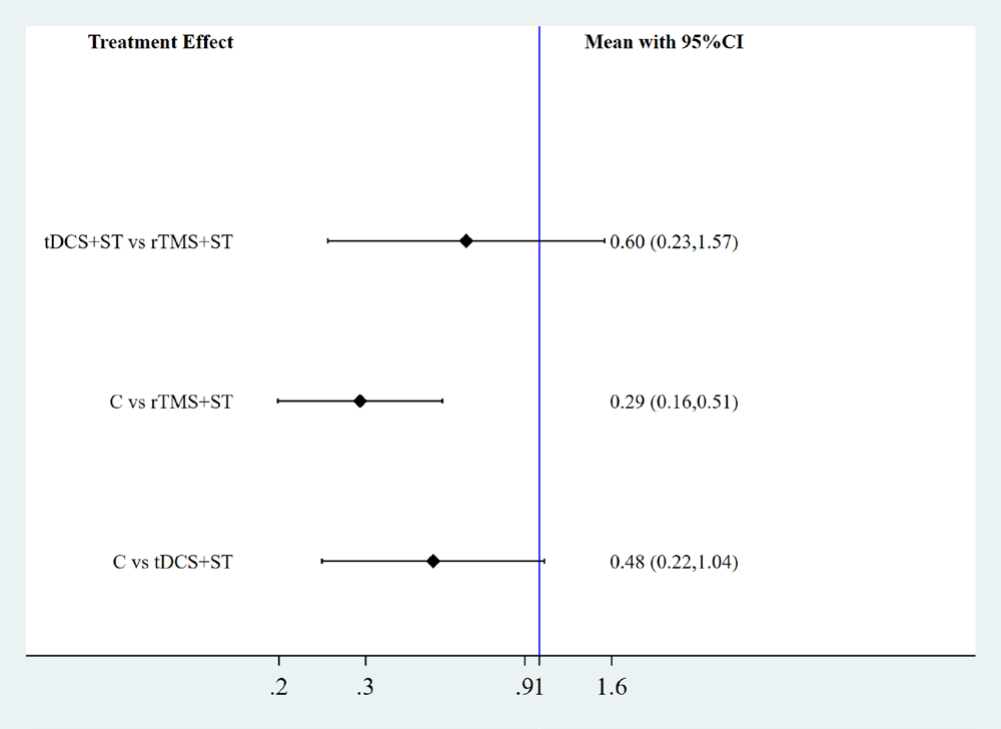
Two-by-two comparison of forest maps (C = control group, rTMS + ST = rTMS + ST group, tDCS + ST = tDCS + ST group). rTMS = repetitive transcranial magnetic stimulation, ST = speech training, tDCS = transcranial direct current stimulation.

**Figure 11.**
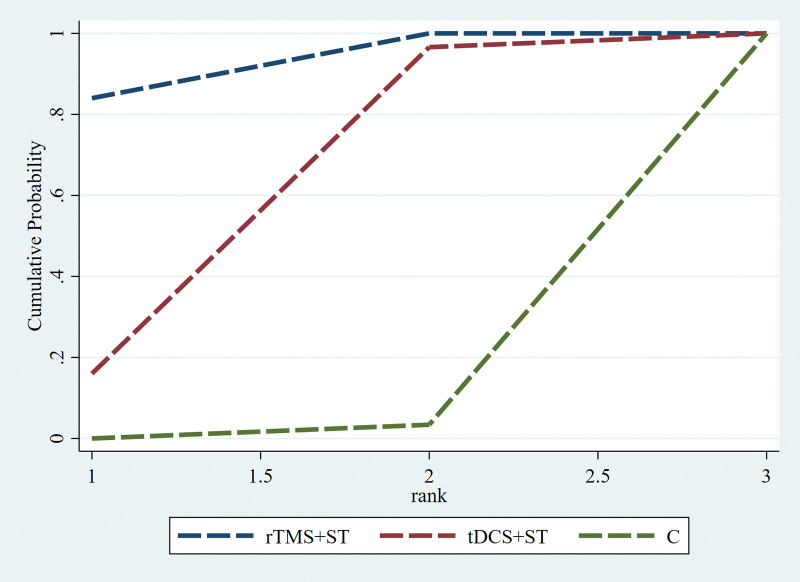
SUCRA probability ranking diagram (C = control group, rTMS + ST = rTMS + ST group, tDCS + ST = tDCS + ST group). rTMS = repetitive transcranial magnetic stimulation, ST = speech training, SUCRA = surface under the cumulative ranking, tDCS = transcranial direct current stimulation.

**Figure 12.**
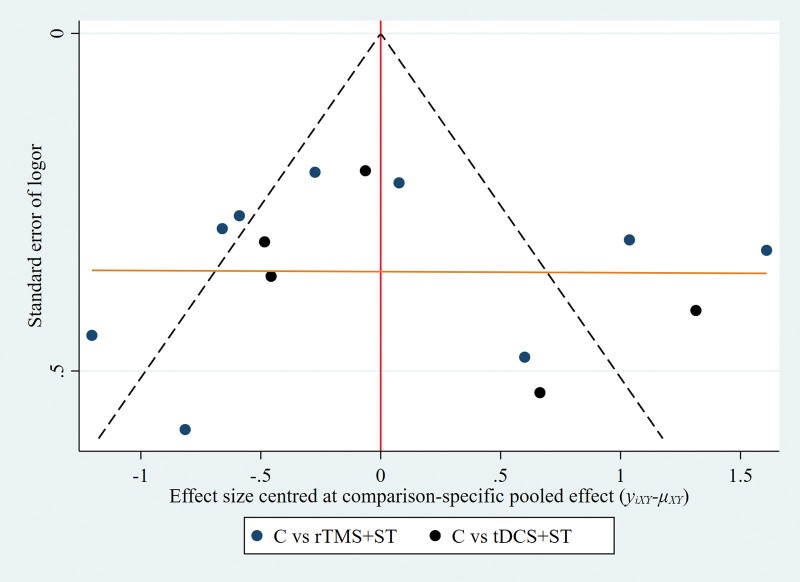
Comparative-corrected inverted funnel plot (C = control group, rTMS + ST = rTMS + ST group, tDCS + ST = tDCS + ST group). rTMS = repetitive transcranial magnetic stimulation, ST = speech training, tDCS = transcranial direct current stimulation.

A Network meta-analysis was performed for the AQ scores, and the evidence network diagram for the effect of the 2 NIBS techniques combined with speech training on the improvement of AQ scores is shown in Figure 9. No closed loops were formed between the interventions; therefore, a consistency model was used for the statistical analyses. The results of the Network meta-analysis showed that the AQ scores of the rTMS + ST group were higher than in the control group, with a statistically significant difference (*P* < .05), and the differences among the remaining groups were not statistically significant (*P* > .05), see Figure 10. The order of probability of SUCRA was: rTMS + ST (92.0%) > tDCS + ST (56.3%) > control group (1.7%), see Figure 11. The results of the comparison-corrected inverted funnel plot show that the scatters are unevenly distributed about the left and right of the X = 0 vertical line, and some of the scatters are in the lower part of the funnel plot, suggesting that there may be a publication bias and a small-sample effect, see Figure 12.

### 3.6. Adverse effects

A total of 6 studies^[21,23–25,27,31]^ demonstrated no significant adverse effects during treatment. In addition, 2 studies^[26,29]^ showed mild adverse reactions, and the treatment measure was tDCS + ST. Adverse reactions mainly included mild dizziness, headache, and pins and needles sensation, which could be relieved by themselves.

### 3.7. Evaluation of the quality of meta-analysis evidence

The results showed that spontaneous speech and listening comprehension scores were intermediate evidence, while AQ scores, repetition scores, naming scores, and clinical efficiency were low evidence, see Table 3.

**Table 3:** Quality grading of meta-analysis outcome indicators.

Outcome indicators	No of studies (No of patients)	Risk of bias	Inconsistency	Indirectness	Imprecision	Publication bias	Certainty
AQ	14 (751)	−1*	−1†	0	0	0	㊉㊉㊀㊀
Spontaneous language	11 (628)	−1*	0	0	0	0	㊉㊉㊉㊀
Listening comprehension	11 (628)	−1*	0	0	0	0	㊉㊉㊉㊀
Repetition	11 (628)	−1*	−1†	0	0	0	㊉㊉㊀㊀
Naming	11 (628)	−1*	−1†	0	0	0	㊉㊉㊀㊀
Clinical efficiency	5 (434)	−1*	0	0	−1‡	0	㊉㊉㊀㊀

AQ = aphasia quotient.

* Bias in allocation concealment and blinding;

† Significant heterogeneity across studies with *I^2^* > 50% and low overlap in the range of confidence intervals.

‡ Sample size < 400 and broad confidence intervals; ㊉㊉㊀㊀ = low evidence; ㊉㊉㊉㊀ = moderate evidence.

## 4. Discussion

PSA is a stroke-induced disruption of the language network between functional language areas or even between the entire cerebral hemispheres, resulting in multifaceted language dysfunction such as phonological, semantic, and syntactic dysfunction.^[10,32]^ As one of the most common complications after stroke, PSA has been gradually recognized and valued by the public and has attracted widespread attention.^[33]^ Currently, conventional speech therapies such as Schulle stimulation, attention training, and music therapy are still the preferred rehabilitation methods.^[34]^ In recent years, some studies have shown that high-intensity speech training is more conducive to the recovery of language function in PSA patients.^[35]^ The limitations of purely conducting speech training, such as short adequate training time, a small number of professional speech therapists, and slow effect, can no longer meet the needs of PSA patients for treatment. Therefore, it is essential to combine other therapies with speech training. NIBS is an emerging noninvasive central neuromodulation technique with the advantages of being noninvasive, having fewer adverse effects, high patient acceptance, and being highly reproducible. Current research favors combining the NIBS technique with speech training for better therapeutic outcomes.

This study systematically reviews the efficacy of the NIBS technique combined with speech training in treating PSA. The results showed that the difference between patients in the NIBS + ST group and the ST group was statistically significant in terms of improving the severity of aphasia, promoting the recovery of language functions such as spontaneous speech, listening comprehension, repetition, and naming, and increasing the clinical efficiency (*P* < .05), suggesting that the addition of the NIBS technique on top of speech training may be better for the recovery of language functions in patients with PSA. Notably, we observed high heterogeneity in AQ and retelling scores. This result may be related to the measurement method of the outcome indicators, the amount of literature included, etc. In addition, there was a difference in the evaluation of tDCS + ST in this study’s traditional meta-analysis and network meta-analysis. In the traditional meta-analysis, the difference in AQ scores between the tDCS + ST group and the ST group was statistically significant (*P* < .05). In contrast, in the reticulated meta-analysis, the difference between the tDCS + ST and ST groups was not statistically significant (*P* > .05). This result may be related to the differences in the duration of the disease, PSA type, and the setting of the treatment parameters of the study subjects. It is still necessary to include many high-quality studies in the future to support whether the effect of tDCS + ST in the treatment of PSA is significant.

The interhemispheric inhibitory and compensatory models have been reported to be the primary theoretical basis for applying the NIBS technique to PSA.^[36]^ The theory suggests that in a healthy state, excitatory or inhibitory activity between the 2 cerebral hemispheres is in a state of equilibrium.^[37]^ The inhibition model assumes that stroke causes a decrease in the excitability of the damaged hemisphere. The balance of mutual inhibition between the hemispheres is disrupted, resulting in a weakening of the inhibitory effect of the damaged hemisphere on the undamaged hemisphere and a relative further increase in the inhibitory effect of the undamaged hemisphere on the damaged hemisphere.^[38]^ In contrast, the compensation model assumes that there is a compensatory mechanism in the undamaged hemisphere, which can replace the damaged region in fulfilling the functions it has lost.^[5]^ Based on these 2 models, the NIBS technique can be used to inhibit nondominant hemispheric language mirror regions or promote dominant hemispheric language-functional regions via electrical or magnetic energy, thereby inducing plastic changes in interhemispheric functional connectivity and facilitating the reconstruction of the language network after stroke.^[3,39]^ Studies have shown that although the NIBS technique significantly improves the outcome of patients with PSA, it is more effective when combined with speech training at the same time.^[40]^ Speech training causes plasticity changes in the language-functional areas of the brain through repeated training, and NIBS technology can directly act on the cerebral cortex to induce plasticity changes in the functional connections of the language-functional areas and distant parts of the brain, thereby enhancing or consolidating the therapeutic effects of speech training,^[41]^ which is consistent with the results of this study.

In the Network meta-analysis, the ordering of SUCRA probability showed that rTMS + ST (92.2%) > tDCS + ST (55.7%), but the difference was not statistically significant (*P* > .05), suggesting that rTMS + ST may be superior to tDCS + ST in improving the severity of aphasia. rTMS and tDCS are the 2 most used NIBS techniques, both of which induce changes in cortical excitability but with different mechanisms of action. rTMS induces a pulsed magnetic field perpendicular to the coil by stimulating the current in the coil, generating small and transient induced currents at relatively shallow subcortical locations to modulate neuronal membrane potentials, thereby temporarily inducing neural excitability in the stimulated region and safely and effectively modulating the functioning of brain circuits.^[42,43]^ tDCS, on the other hand, modulates cortical excitability by applying a weak direct current continuously to the scalp via electrode sheets (cathode and anode), which causes changes in neuronal membrane potentials in the cortical region under the electrode sheets.^[44,45]^ It has been reported that tDCS is less powerful and produces weaker stimuli than rTMS, which may contribute to the superiority of rTMS over tDCS in improving aphasia severity in PSA patients.^[46]^

Currently, the stimulation protocols of the NIBS technique for PSA include; Excitatory stimulation of the region surrounding the speech-dominant hemispheric lesion; Inhibitory stimulation of the nonspeech-dominant hemispheric region, and; Combined stimulation of the above 2 modalities.^[47]^ When treating PSA by NIBS technique in clinical therapy, the optimal stimulation protocol needs to be selected according to the patient’s situation, such as stroke type, lesion site, aphasia type, and whether it is tolerated or not. In addition, treatment parameters such as stimulation site, frequency, intensity, and duration are also important factors affecting the therapeutic effect.^[48]^ In the rTMS studies included in this study, low-frequency stimulation (≤ 1 Hz) was predominant. Only 1 study had high-frequency stimulation (> 1 Hz), and the coil was primarily placed in the right mirror area of speech function when low-frequency stimulation was performed. In contrast, the coil was placed in the left area of speech function when high-frequency stimulation was performed, with stimulation intensities ranging from 80% to 100% of the threshold of motor-evoked potentials and treatment periods ranging from 2w to 8w. In the tDCS studies, 2 were anodic stimulation, and the remaining 4 were bilateral stimulation, with a current of 1.2 mA, a unilateral treatment time of 20min, and a treatment period of 2 w to 6 w. The treatment protocols were relatively similar. More large-sample, high-quality studies are needed to investigate whether there are differences in the effects of NIBS techniques with different therapeutic parameters in patients with PSA and the selection of the optimal regimen.

This study still has some limitations. First, some of the interventions included a small number of studies with low sample sizes, such as the high-frequency r TMS treatments, which may affect the reliability of the outcome comparisons. Second, the overall quality of the included studies could have been better, with most not reporting allocation concealment and implementation of blinding and only 5 studies using sham stimulation in the control group. The rest treated the control group with speech training alone, with a more pronounced difference in intervention modalities between the 2 groups, which may have influenced the implementation of blinding. Finally, differences in the type of aphasia of patients, duration of treatment, and site of stimulation between studies are likely to create a risk of bias in the results.

## 5. Conclusion

Based on the current evidence, this study concluded that the NIBS technique combined with speech training could effectively improve the severity of aphasia in PSA patients, promote the recovery of language functions such as spontaneous speech, listening comprehension, repetition, and naming, and improve the clinical efficiency of PSA, and recommended that rTMS combined with speech training should be given priority in the clinical treatment of PSA. Given the limitations of this study, the above conclusions need to be verified by further high-quality experiments.

## Author contributions

**Conceptualization:** Congli Han, Jienuo Pan, Nan Wang.

**Data curation:** Congli Han, Jienuo Pan, Nan Wang.

**Formal analysis:** Congli Han, Bingshun Tang, Tao Han.

**Methodology:** Congli Han, Jiqin Tang, Bingshun Tang, Tao Han.

**Software:** Congli Han, Jiqin Tang, Jienuo Pan, Nan Wang.

**Validation:** Congli Han, Jiqin Tang, Bingshun Tang, Tao Han.

**Writing – original draft:** Congli Han, Jiqin Tang.

**Writing – review & editing:** Congli Han, Jiqin Tang.
